# A mechanism study of DUSP1 in inhibiting malignant progression of endometrial carcinoma by regulating ERK/AP-1 axis and dephosphorylation of EPHA2

**DOI:** 10.7150/jca.81069

**Published:** 2023-02-27

**Authors:** Shu Lei, Xiangjun He, Xiao Yang, Xuan Gu, Yijiao He, Jianliu Wang

**Affiliations:** 1 Department of Gynecology and Obstetrics, Central Laboratory & Institute of Clinical Molecular Biology, Peking University People's Hospital, No.11 Xizhimen South Street, Beijing 100044, China.; 2 Department of Gynecology and Obstetrics, The Third Affiliated Hospital of Zhengzhou University, No.3 Kangfu Middle Street, Zhengzhou 450052, China.

**Keywords:** Endometrial carcinoma, DUSP1, AP-1 complex, MAPK pathway, EPHA2.

## Abstract

**Background:** Endometrial carcinoma is one of the most common female malignancies worldwide. Based on our preliminary investigation, DUSP1 was identified as a potential biomarker for endometrial carcinoma prognosis, but its function and mechanism remained unclear.

**Methods:** In this study, genes highly correlated with DUSP1 in endometrial cancer were found through correlation analysis, and the promoter sequence of DUSP1 was analyzed by PROMO program. Next-generation phosphorylation mass spectrometry was used to explore new downstream target proteins and pathways of DUSP1 in endometrial carcinoma. The mRNA and protein expression levels were detected by real-time quantitative PCR, immunohistochemistry and Western blotting. The cell survival and proliferation were analyzed by CCK8 assay, cell apoptosis was analyzed by Annexin-V-APC and PI dual staining assay, and the cell invasion was analyzed by Transwell method.

**Results:** (1) There was a high correlation between the expression of DUSP1 and the genes involved in AP-1 complex and its co-expression network. (2) Promoter sequence analysis predicted that the members of AP-1 complex might be the upstream transcriptional regulators of DUSP1. (3) Transfection experiments proved DUSP1 can inhibit tumor growth and invasion, and promote apoptosis by regulating ERK pathway. (4) The results of phosphorylation mass spectrometry showed that overexpression of DUSP1 mainly dephosphorylated EPHA2 in endometrial carcinoma, and co-immunoprecipitation verified the protein interaction between DUSP1 and EPHA2. (5) Overexpression or knockdown of EPHA2 significantly changed the phosphorylation level of EPHA2. (6) The expression of EPHA2 protein was high in patients with more aggressive endometrial cancer. (7) Using EPHA2 inhibitor could significantly slow down the growth rate of tumor cells.

**Conclusion:** (1) There exists a mutual regulation relationship between DUSP1 and AP-1 co-expression network in endometrial carcinoma. (2) It is reported for the first time that DUSP1 phosphatase acts on the ser899 site of EphA2 in endometrial carcinoma. (3) DUSP1 can inhibit tumor growth and invasion, and promote apoptosis by regulating MAPK pathway through directly dephosphorylating ERK, or by dephosphorylating EPHA2.

## Introduction

Endometrial carcinoma (EC) is the most common gynecological malignancy in the developed world, second only to cervical cancer in developing countries. Although 80% of EC patients are typically diagnosed at an early stage, 20% have a worse prognosis 1. The traditional classification of EC was established by Bohkman 2, but due to the heterogeneity of EC, research continues for biomarkers to define the pathogenesis, progression, prognosis, and set the stage for targeted therapeutics.

Our previous study focused on genes related to prognosis of endometrial carcinoma. A high-throughput gene chip, including prognosis-related genes selected from NCBI (National Center for Biotechnology Information) and CNKI (China National Knowledge Infrastructure), was designed and used for biomarkers screening in EC patients from Peking University People's Hospital. DUSP1 (dual specificity protein phosphatase 1) was identified as a potential positive prognostic molecular marker 3. Subsequent immunohistochemical detection of DUSP1 in endometrioid adenocarcinoma found that there was a significant decrease of DUSP1 expression in more aggressive subtypes 4. DUSP1 plays different roles in different kinds of tumors. In hepatocellular carcinoma 5, prostate cancer 6, head and neck squamous cell carcinoma 7 as well as other tumor types, DUSP1 was significantly down-regulated in aggressive phenotypes. In contrast, in non-small-cell lung cancer 8 and pancreatic cancer 9, DUSP1 was found to promote angiogenesis, invasion and metastasis. However, there have been few studies that have investigated the function and mechanism of DUSP1 in EC.

DUSP1 is a major dual-specificity protein phosphatase of the DUSP family that dephosphorylates both the threonine/serine and tyrosine residues. Members of the DUSP family play a critical role in controlling MAPK (mitogen activated kinase-like protein) signaling and DUSP1 dephosphorylates 3 major MAPK subfamilies MAPK/JNK, MAPK/p38, and MAPK/ERK 10. It targets specific MAPK pathways in different types of tumors. For example, it efficiently dephosphorylates JNK pathway in prostate cancer 11, but acts on p38/MAPK pathway in hepatocellular carcinoma 12.

In the present study, we aimed to investigate how DUSP1 is regulated, what other target proteins it modifies, and by what mechanism it affects tumor progression. We demonstrated that DUSP1 influences the expression of AP-1 (activator protein 1) and its co-expression network through dephosphorylation of ERK/MAPK, and AP-1, in turn, can regulate the expression of DUSP1, forming an indirect positive feedback loop. We also proved EPHA2 is an important new target of DUSP1 and EPHA2 also plays an important role on tumor growth of EC.

The AP-1 (activator protein 1) transcription factor is a dimeric complex that consists of members of the JUN, FOS, ATF (activating transcription factor) and MAF (musculoaponeurotic fibrosarcoma) protein families 13. It was reported that in breast cancer AP-1 transcription factor components, i.e., JUN, JUNB, FOS, FOSB, in addition to DUSP1, EGR1, NR4A1, IER2 and BTG2, behave as a conserved co-regulated network 14, most of which are transcriptional factors. Kesarwani found c-Fos and DUSP1 deficiency alters the AP-1 regulated networks 15. Due to the high correlation between AP-1 related genes and DUSP1, we speculated that there might be a regulatory relationship between them.

Ephrin type-A receptor 2 (EPHA2) is an important member of the large receptor tyrosine kinase (RTK) family 16, which is composed of 976 amino acids, including extracellular domain, transmembrane domain containing ligand binding domain and intracellular domain containing tyrosine kinase domain 17. Recent studies have shown that EPHA2 is highly expressed in a variety of tumors and is closely related to the prognosis of tumor patients. EPHA2 participates in the interaction with other membrane receptors and can affect the downstream RAS/PI3K/AKT and RAS/MAPK signaling pathways. However, there has been no study involving the regulation relationship of EPHA2 and DUSP1 in any tumor.

## Methods and Materials

### Antibodies, primers, siRNAs, and plasmids

The antibodies against DUSP1 (#2857S), ERK1/2 (#4695S), p-ERK1/2 (4370S), EPHA2 (6997S), GAPDH (#2118S) were all purchased from CST (Cell Signaling Technology, Danvers, USA) and antibodies against JNK (#51151-1-AP) and p-JNK (#80024-1-RR) were purchased from ProteinTech (Rosemont, USA). SiRNAs of DUSP1 and EPHA2 were synthesized from GenePharma Co. Ltd (Suzhou, China). Primers of genes for qPCR amplification were all synthesized by Tsingke Biotechnology Co. Ltd (Beijing, China). Plasmid PC-h-DUSP1 for DUSP1 overexpression and plasmid pcDNA3.1-EPHA2 for EPHA2 overexpression were all designed and synthesized by Hanbio Biotechnology Co. Ltd (Shanghai, China). Sequences of primers and siRNAs were all listed in **Table 1**.

### Cell culture, transfection, and drug treatment

Ishikawa, HEC-1B and HEC-50B human EC cell lines were all purchased from JCRB cell bank (Tokyo, Japan) and cultured according to manufacturer's instructions. Ishikawa is highly differentiated, HEC-1B is moderately differentiated and HEC-50B is poorly differentiated. For knockdown and overexpression experiments, cells were transfected with Lipofectamine 3000 (Thermo Fisher Scientific). We used cells transfected with blank vector plasmid as negative control group. T-5224 (AP-1 inhibitor, Catalog No. B4664) was purchased from APExBIO (Houston, USA).

### RNA extraction, reverse transcription, and real-time PCR

Total RNA was isolated from 3 EC cell lines with different treatment using the RNeasy Mini Kit (Qiagen, Hilden, Germany). Then 1.5 µg total RNA was reverse transcribed using Hifair^®^ Ⅱ 1st Strand cDNA Synthesis Kit (Yeasen, Shanghai, China). The cDNAs were used for real-time PCR amplification using Hieff^®^ qPCR SYBR Green Master Mix (Yeasen, Shanghai, China).

### Apoptosis assay

Cell apoptosis was evaluated by flow cytometry using an Annexin V-APC/PI Apoptosis Detection Kit (KeyGen BioTECH, Nanjing, China). Cells with different treatment were incubated in a 6-well plate for 48 hours. All cells were trypsinized and the resuspended cells were washed twice with PBS. Cells were then resuspended in 500 µL binding buffer, and 5 μL Annexin V-APC and 5 μL propidium iodide (PI) were added into cell suspension at room temperature for 15 min in the dark before detection.

### Cell proliferation assay

We seeded 5*10^3^ cells into 96 well plates with 100 μL complete medium. After incubation for 6, 24, 48 and 72 hours, cell viability was detected via CCK8 kit (Soloarbio, Beijing, China) according to manufacturer's instructions. Results were assessed with microplate reader at 450 nm. Triplicating assay was performed.

### Cell invasion assays

The suspension of three different cell lines (1 × 10^5^ cells) were seeded into Matrigel coated transwell inserts (Corning, USA, Cat log: #354480#) with a polyethylene terephthalate membrane pore size of 8 μm in 24-well plates. Cells at upper chambers were maintained in a culture medium containing 10% FBS, and similar medium without FBS were put in lower wells. After incubation at 37℃ for 48h, cells were fixed with 4% paraformaldehyde for 20min at room temperature, and stained with crystal violet for 30mis (Solorbio, Beijing, China). Cells that did not migrate across the transwell membrane were then removed by gently wiping with a cotton swab. The invasion of cancer cells was observed by reversed microscope.

### Western Blotting

Whole cell lysates of 50 μg protein were electrophoresed through 10% acrylamide Tricine-SDS gels. Then the proteins were transferred onto polyvinylidene difluoride membranes (Millipore, Darmstadt, Germany). Blocked in 5% skim milk for 1 hour at room temperature, the membranes were incubated with primary antibodies at 4°C overnight, followed by incubation using anti-rabbit or mouse horseradish peroxidase-conjugated secondary antibody (ZSGB-BIO, Beijing, China) at room temperature for 1 h. The signal was visualized using Immobilon Western HRP Substrate (Millipore, Darmstadt, Germany).

### Phosphorylation mass spectrometry and bioinformatic analysis

SDT (4%SDS, 100mM Tris-HCl, 1mM DTT, pH7.6) buffer was used for sample lysis and protein extraction. The digest peptides of each sample were desalted on C18 Cartridges, concentrated by vacuum centrifugation and reconstituted in 40 µL of 0.1% formic acid. The enrichment of phosphopeptides was carried out using High-Select^TM^ Fe-NTA Phosphopeptides Enrichment Kit (Thermo Scientific). After being lyophilized, the phosphopeptides peptides were resuspended in 20 µL loading buffer (0.1% formic acid).

LC-MS/MS analysis was performed on a timsTOF Pro mass spectrometer (Bruker) that was coupled to Nanoelute (Bruker Daltonics) for 60 min. The peptides were loaded on a C18-reversed phase analytical column in 0.1% formic acid and separated with a linear gradient of buffer mixed with 84% acetonitrile and 0.1% formic acid at a flow rate of 300 nl/min. The mass spectrometer was operated in positive ion mode. The quantitative information of the target protein set was normalized and then, R (version 3.4) was used to classify the two dimensions of sample and protein expression at the same time, and finally a hierarchical clustering heat map was generated.

### Co-Immunoprecipitation (Co-IP)

We applied co-immunoprecipitation (Co-IP) to verify whether there is an interaction between the two proteins using Pierce^TM^ classic magnetic Co-IP kit (Thermo Fisher, USA). Ice-cold IP Lysis Buffer was added in EC cells for protein lysis and extraction. The antibody against DUSP1 (#sc-373841, Santa Cruz, USA) was then added to the sample to form an immune complex. The complex was incubated overnight and then washed to remove non-bound material. Finally, low-pH elution buffer dissociated the bound immune complex from the Protein A/G.

### Immunohistochemistry (IHC)

46 tumor tissue specimens obtained from Peking University People's Hospital were fixed with 4% formalin and paraffin embedded before cutting it into 4 μm thick sections. Tissue slides were incubated overnight with primary antibodies. After PBS washes for three times, the sections were incubated with secondary antibody (ZSGB-BIO, Beijing, China) for 30 mins at room temperature. The slides were then stained with DAB substrate and counterstained with hematoxylin. The study was approved by the Ethics Committee of the People's Hospital, Peking University.

### Statistics and public data resource

Statistical analysis was performed using GraphPad Prism software (v.9.0; GraphPad Software, Inc.). The student's t‑test and Mann‑Whitney U‑test were used to compare the means between two groups, and One-way ANOVA was used for comparisons among three or more groups. P<0.05 was considered to indicate a statistically significant difference.

Gene co-expression data (177 samples) were retrieved from cBioPortal [www.cbioportal.org; Dataset, Uterine Corpus Endometrial Carcinoma (TCGA, Firehose Legacy)]. Then the mRNA expression data of 543 patients were used for scatter plot drawing, which was sorted out from dataset downloaded from https://xenabrowser.net/datapages/ (version 2019) after excluding repetitive specimens, recurrent samples, and normal solid tissue samples.

## Results

### The expression of DUSP1 was highly correlated with genes affiliated to AP -1 network

**Figure 1:**
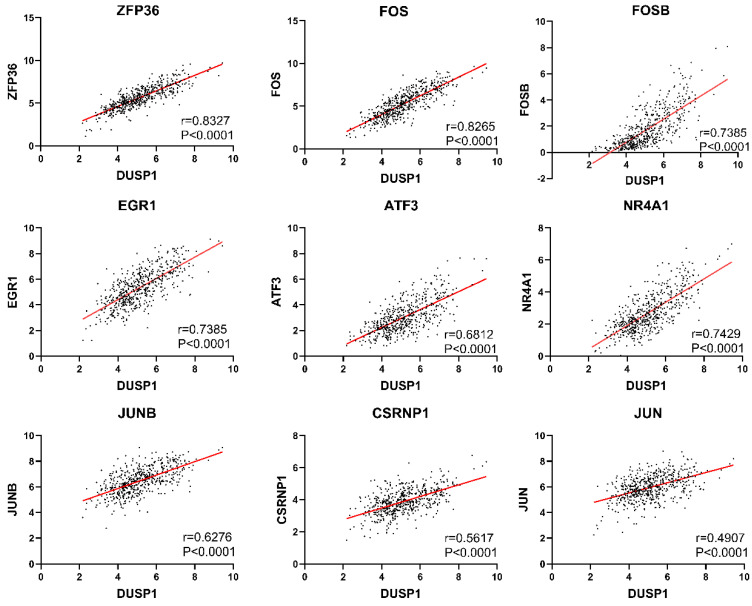
** Correlation analysis of DUSP1 in endometrial carcinoma.** Genes with the highest correlation with DUSP1 in EC were selected in this study. Individual Pearson coefficient and P values were marked on the figures.

Because the genes with direct upstream and downstream regulation are usually highly correlated in expression level, we used the “co-expression” tool of cBioPortal (http://www.cbioportal.org/) to retrieve genes correlated with DUSP1. We selected genes with Spearman's correlation coefficient greater than 0.5 (**Table 2**) and found that many of them belong to AP-1 complex related genes. As the “co-expression” tool only showed a result of 177 samples, we used the mRNA expression data of 543 samples from TCGA to verify the correlation and plotted the scatter distribution diagram. The scatter plots of these genes with trend line and correlation coefficient were shown in **Fig. 1.**

### Mutual regulation existed between DUSP1 and AP-1 related genes

#### Modulating the expression level of DUSP1 affected the expression of AP-1 associated genes

**Figure 2:**
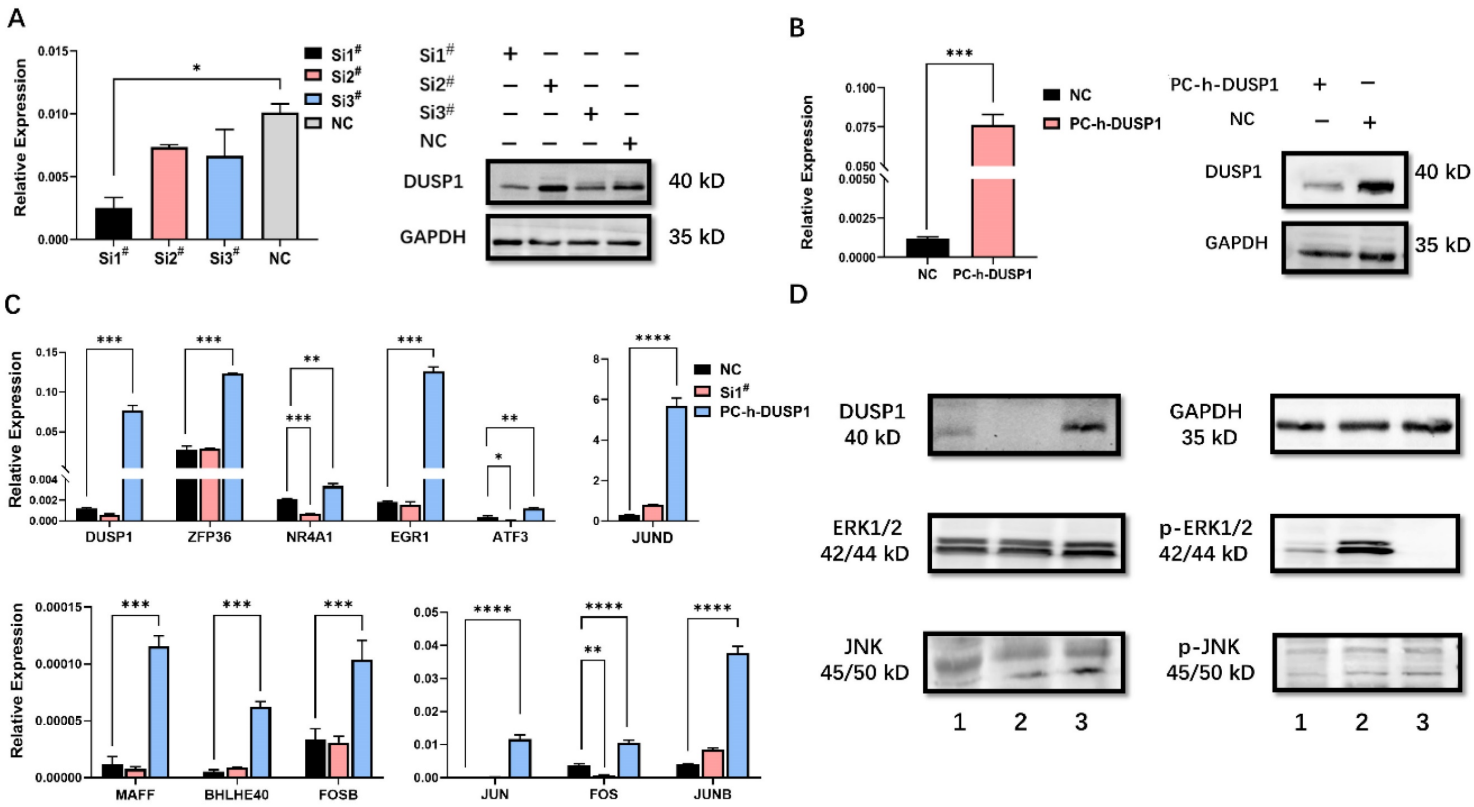
** Modulation of DUSP1 affects the expression of AP-1 genes. (A)** mRNA and protein expression level after transfection with different DUSP1 siRNAs. **(B)** mRNA and protein expression level after transfection with plasmid PC-h-DUSP1. **(C)** Expression of selected genes in Ishikawa cells after transfection of DUSP1.** (D)** Overexpression of DUSP1 dephosphorylate MAPK pathway. 1: negative control group (NC), 2: knockdown of DUSP1 in Ishikawa 3: overexpression of DUSP1 in Ishikawa; Relative expression: the relative expression calculated based on ΔCT method; Si1^#^: DUSP1 SiRNA1; Si2^#^: DUSP1 SiRNA2; Si3^#^: DUSP1 SiRNA3; PC-h-DUSP1: plasmid PC-h-DUSP1 for overexpression; NC: negative control; *: p<0.05; **: p<0.01; ***: p<0.001; ****: p<0.0001.

The efficiency of knockdown and overexpression of DUSP1 in EC cells was evaluated by qPCR and WB (**Fig. 2A, B**). Si1^#^ was selected for subsequent RNA interference. By analyzing genes with high correlation coefficient with DUSP1 expression, AP-1 constituent members and AP-1 co-expression network genes were selected to investigate the effects of up- and down-regulated DUSP1 on their expression level. DUSP1 overexpression increased the mRNA levels of all of these genes significantly. Knockdown of DUSP1 decreased the expression levels of NR4A1, ATF3, FOS significantly (**Fig. 2C**), while the expression of ZFP36, EGR1, JUND, MAFF, BHLHE40, FOSB, JUN, and JUNB were similar to that of the control group. These inconsistent results may be attributed to the feedback regulation between these genes, resulting in the compensatory increase of the expression of these genes in DUSP1 knockdown cells, or the off-target effects caused by siRNA 18.

#### DUSP1 modulates the expression of AP-1 related genes by dephosphorylating ERK

Because DUSP1 is not a transcription factor but rather a phosphatase, it is impossible for DUSP1 to directly regulate the expression of AP-1 complex members and their co-expression network genes. Previous studies have shown that the expression of AP-1 is regulated by the MAPK pathway 19-21 and DUSP1 can inhibit the biological activity of three major MAPK pathways by dephosphorylation. As DUSP1 could target a specific MAPK pathway in different kinds of tumors, we conducted WB assay to find out the main target of the MAPK pathway of DUSP1. The level of phosphorylated ERK was significantly up- or down-regulated in EC cells by RNA interference or overexpression of DUSP1, respectively (**Fig. 2D**), indicating that the ERK pathway was the main dephosphorylation target of DUSP1.

#### AP-1 inhibitor changed the expression of DUSP1

**Figure 3:**
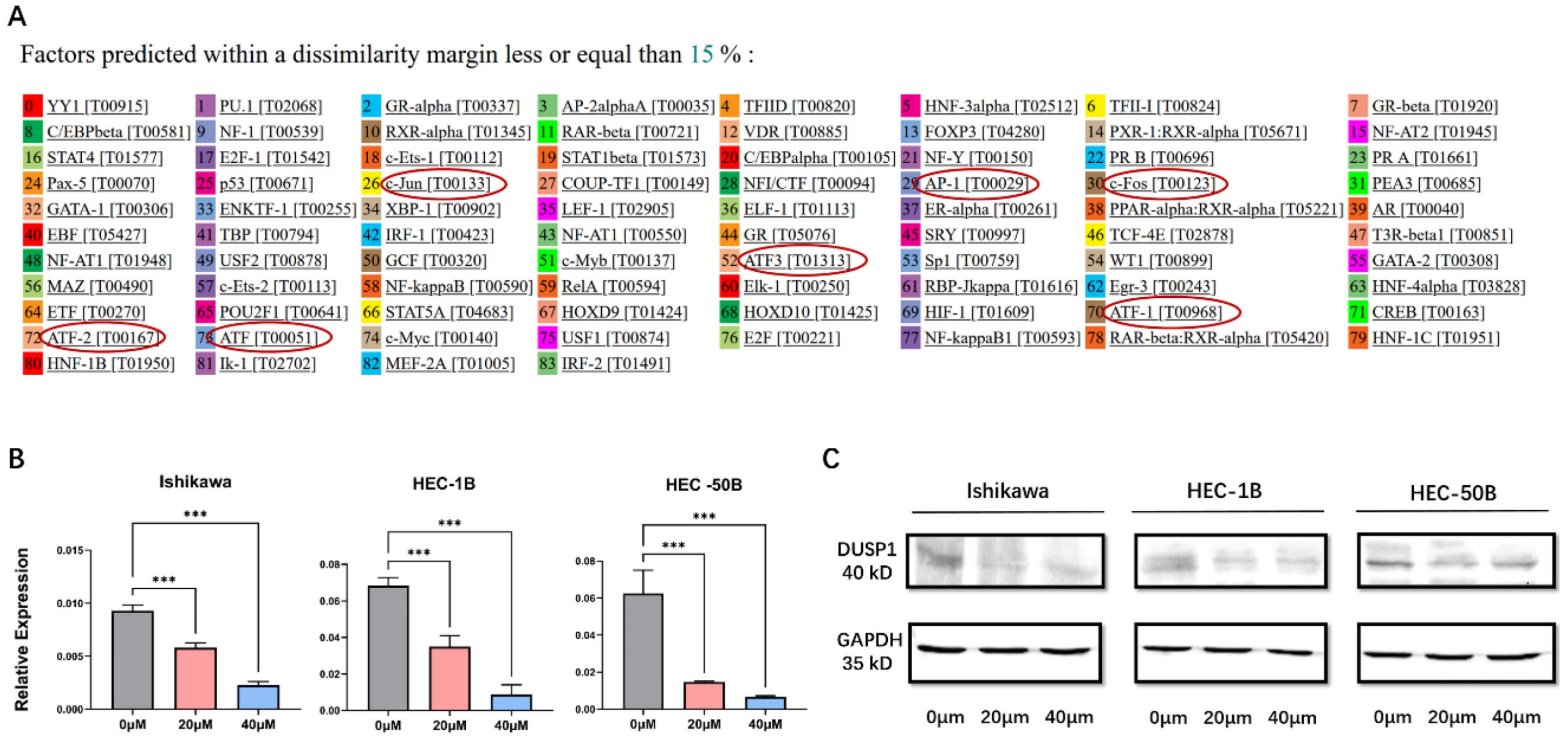
** AP-1 inhibitor could affect the expression of DUSP1. (A)** Prediction of transcription factor binding sites on the promoter of DUSP1. **(B)** mRNA expression of DUSP1 after using different concentrations of AP-1 inhibitor. **(C)** protein expression of DUSP1 after using different concentrations of AP-1 inhibitor. Relative expression: the relative expression calculated based on ΔCT method; ***: p<0.001.

To explore the upstream genes regulating the expression of DUSP1, the PROMO program (http://alggen.lsi.upc.es) was used to predict the binding sites of transcription factors on the promoter of DUSP1. After limiting the species to human and the dissimilarity margin range to 15% in the software setting, multiple binding sites of the AP-1 complex on the promoter of DUSP1 were identified (**Fig**.**3A**), including AP-1, c-Jun, ATF, ATF1, ATF2, ATF3. We speculated that that AP-1 might be the upstream transcriptional factor of DUSP1 and might regulate the expression of DUSP1 in turn.

We added AP-1 inhibitor T-5224 to three kinds of EC cells with different characteristics at different concentrations. The mRNA and protein expression of DUSP1 decreased significantly with the increase of inhibitor concentration (**Fig. 3B, C**).

Therefore, the results indicated that DUSP1 could regulate the expression of AP-1 related genes, and AP-1, in turn, affected the expression of DUSP1, forming a positive feed-back loop.

### Overexpression or knockdown of DUSP1 changed the biological characteristics of EC cells

#### DUSP1 knockdown and overexpression changed cell apoptosis ratio

**Figure 4:**
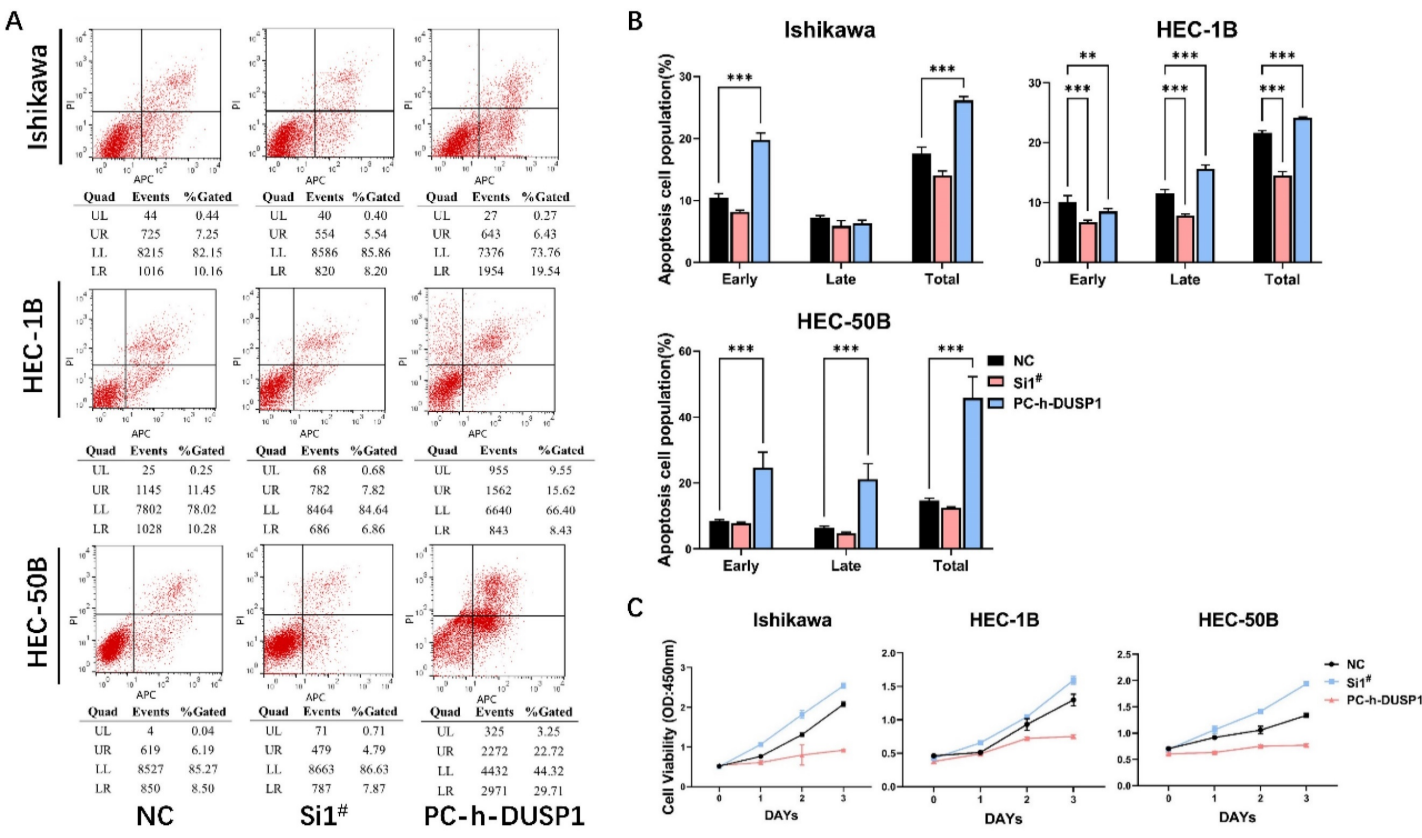
** Transfection of DUSP1 altered the cell apoptosis and viability of EC cell lines. (A)** Cell apoptosis of different EC cell lines after DUSP1 transfection. **(B)** Statistics of apoptosis after transfection. **(C)** Proliferation ability of three kinds of EC cells transfected with DUSP1. Si1^#^: DUSP1 SiRNA1; PC-h-DUSP1: plasmid PC-h-DUSP1 for overexpression; NC: negative control; **: p<0.01; ***: p<0.001.

The apoptosis results of the three EC cell lines were similar. Following overexpression of DUSP1, the percentage of normal cells decreased, and the percentage of apoptotic cells increased significantly, and the number of dead cells in HEC-1B and HEC-50B cell lines increased significantly. After down-regulation of DUSP1 expression, the percentage of apoptotic cells decreased, and the proportion of normal cells increased (**Fig. 4A, B**).

#### Overexpression or knockdown of DUSP1 suppressed the growth of EC cells

CCK-8 assays were used to detect the viability of EC cells (Ishikawa, HEC-1B, HEC-50B) following knockdown or overexpression of DUSP1. Compared with the growth rate of normal endometrial cancer cells as the control, DUSP1 overexpression was found to significantly inhibit the growth of endometrial cancer cells, whereas the knocking down of DUSP1 produced the opposite result. On the third day, overexpression of DUSP1 indicated a trend toward growth stagnation in the endometrial cancer cells, especially in HEC-1B and HEC-50B (**Fig. 4C**).

#### Modulation of the expression level of DUSP1 changed the invasion ability of endometrial carcinoma

**Figure 5:**
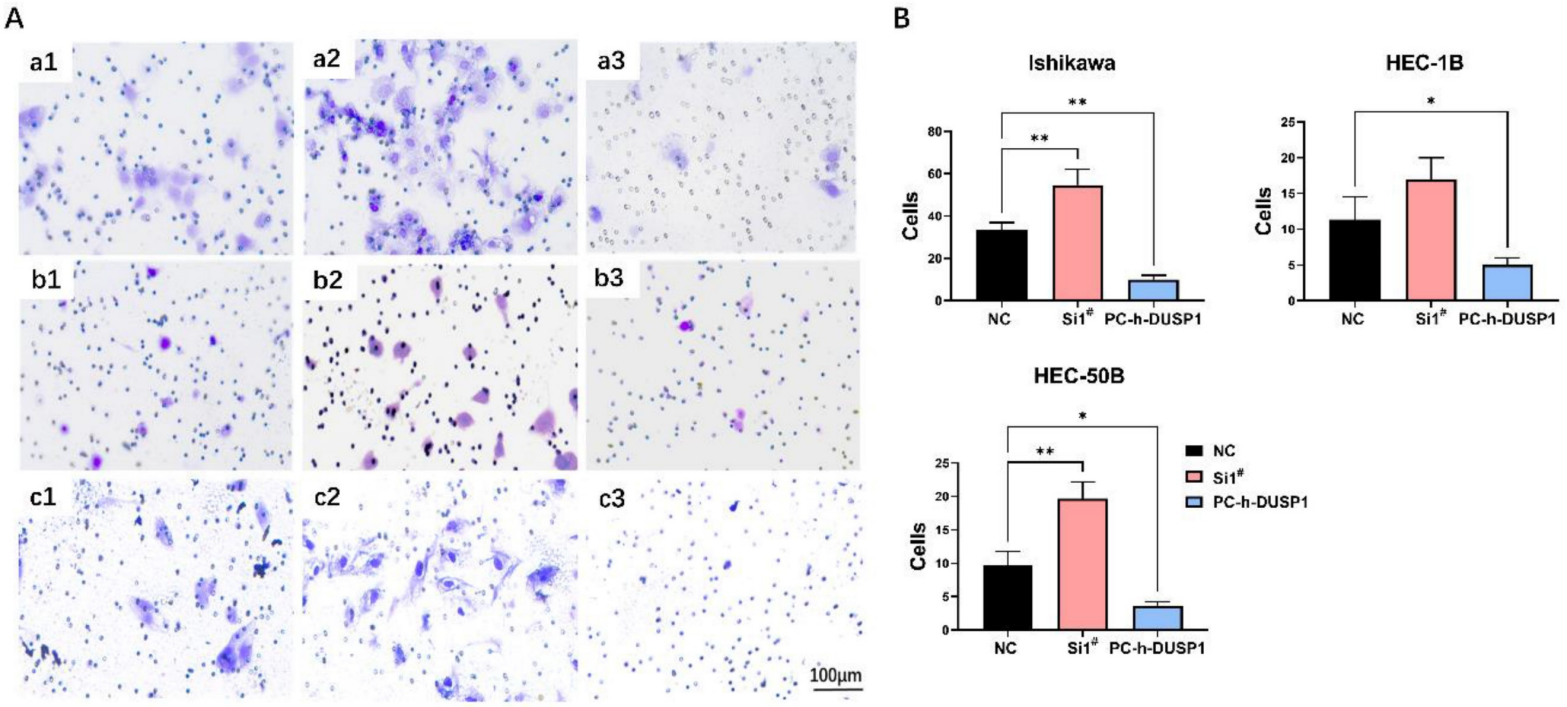
**Regulation of DUSP1 changed the invasion ability of endometrial carcinoma. (A)** Representative pictures of transwell invasion assay after DUSP1 transfection. **a1~a3**: control group, knockdown of DUSP1 and overexpression of DUSP1 in Ishikawa cell line; **b1~b3**: control group, knockdown of DUSP1 and overexpression of DUSP1 in HEC-1B cell line; **c1~c3**: control group, knockdown of DUSP1 and overexpression of DUSP1 in HEC-50B cell line. The scale bar represents 100 μm. **(B)** The number of transmembrane cells.

Transwell invasion assay showed that DUSP1 could affect the capability of tumor invasion in EC. The number of trans-membrane EC cells increased in the DUSP1 deficiency group compared to control group, and the number drastically decreased in the DUSP1 overexpression group (**Fig. 5A, B**). In Ishikawa cells, the average number of normal untreated cells passing through Matrigel in the same size field was 34, which increased to 55 following DUSP1 knockdown, and only 7 after DUSP1 overexpression; in HEC-1B cells, the corresponding numbers were 11, 17, and 5, respectively; and in HEC-1B cells, the numbers were 9, 20, and 4, respectively.

### Phosphorylation mass spectrometry experiments identified EPHA2 as an important target of DUSP1

**Figure 6:**
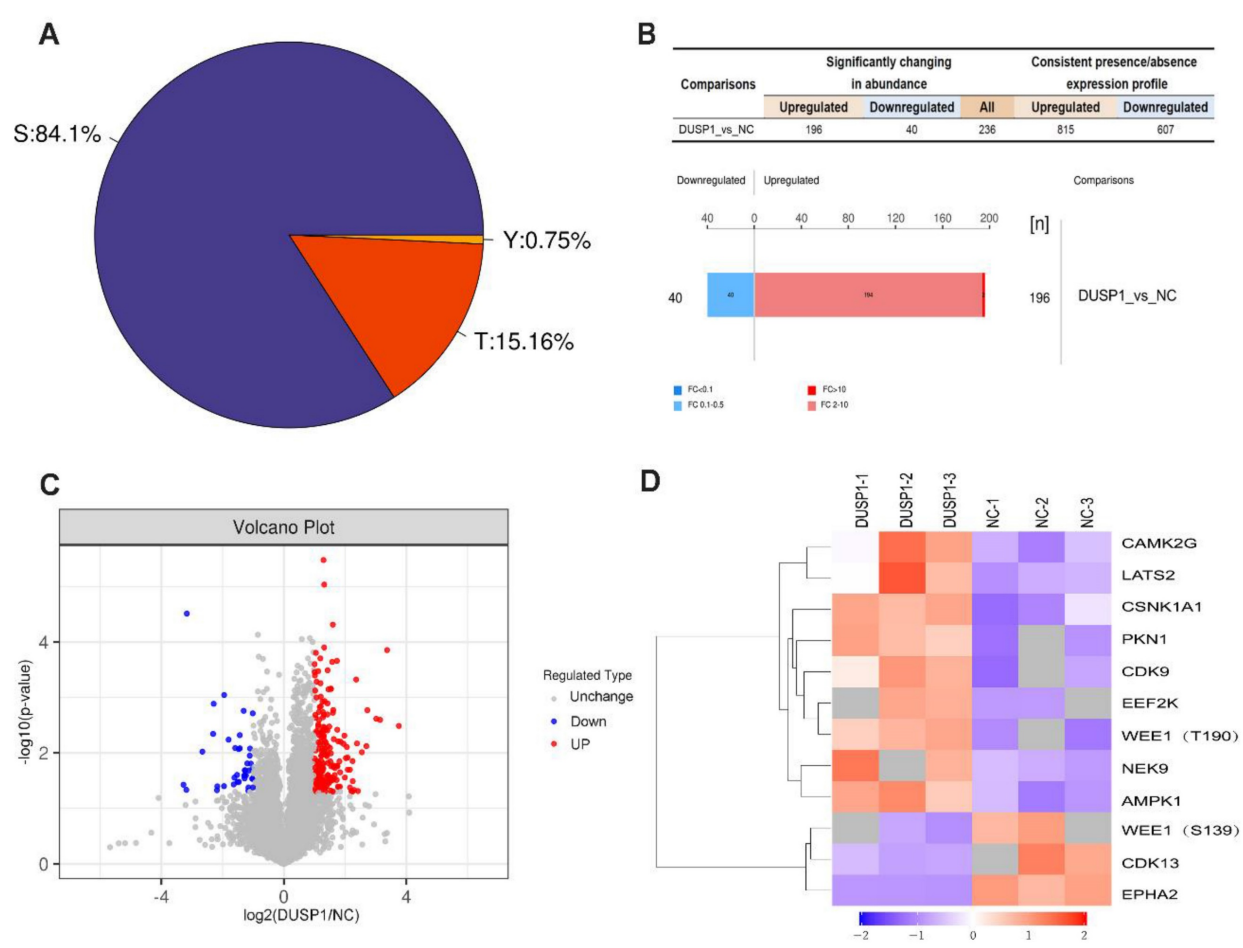
** Phosphorylation omics analysis after DUSP1 overexpression. (A)** Distribution of phosphorylation amino acids of DUSP1. S: serine; T: threonine; Y: tyrosine. **(B)** Statistics and histogram of quantitative difference of phosphorylated peptides. **(C)** Volcano plot. **(D)** Main phosphorylated or dephosphorylated kinases after DUSP1 overexpression. NC: negative control.

In order to further study the effect of DUSP1 on the modification of its downstream target proteins in EC, our research group adopted the phosphorylation 4D label-free quantitative proteomics technology to obtain the difference in phosphorylation modification caused by overexpression of DUSP1. **Figure 6A** shows the distribution proportion of phosphorylation sites on serine (Ser), threonine (Thr) and tyrosine (Tyr). Among them, the largest number of phosphorylation changes in amino acids was found in serine, accounting for 84.1%, threonine for 15.16%, and tyrosine only for 0.75%. The experimental data were further screened for differential expression to find out the target dephosphorylation downstream of DUSP1 in EC. Taking the expression fold (FC) > 2.0 times (up-regulation more than 2.0 times or down-regulation less than 0.5 times) and P value < 0.05 (t-test test) as the screening criteria, 236 were significantly different in the change of phosphorylation modified peptides, 196 were up-regulated and 40 were downregulated (**Figure 6B, C**). After systematic analysis, the most significant difference in dephosphorylation genes in EC after DUSP1 overexpression is EPHA2, and the target site is Ser899 (**Figure 6D**).

### Regulatory relationship between DUSP1 and EPHA2

**Figure 7:**
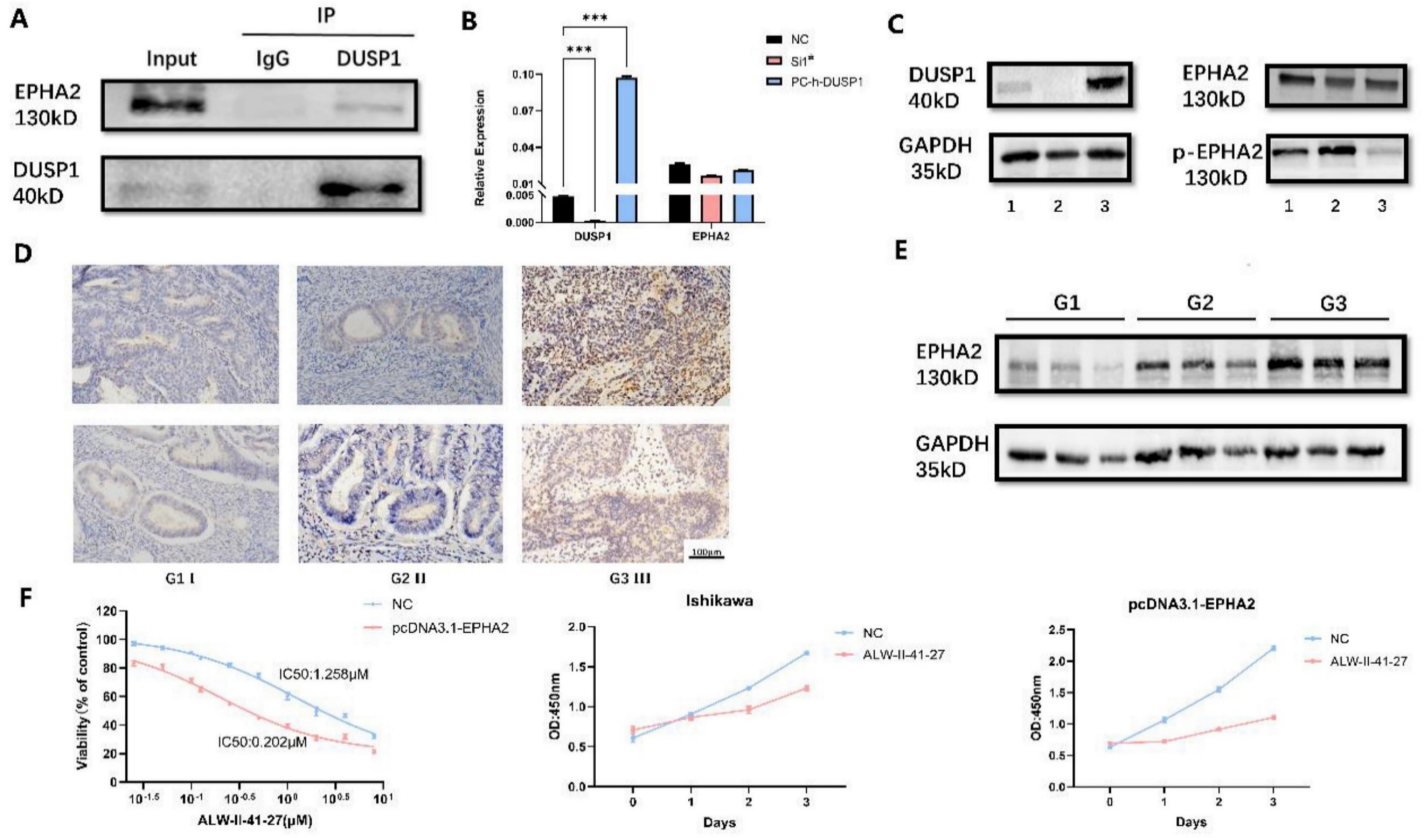
** Effect of EPHA2 on tumor growth in endometrial carcinoma. (A)** Co-IP detection of DUSP1 and EPHA2 **(B)** mRNA expression of EPHA2 after DUSP1 transfection. Relative expression: the relative expression calculated based on ΔCT method; **: p<0.01; ***: p<0.001. **(C)** protein expression of EPHA2 after DUSP1 transfection. 1: negative control group; 2: knockdown of DUSP1 in Ishikawa cell line; 3: overexpression of DUSP1 in Ishikawa cell line. **(D)** Representative pictures of EPHA2 expression detected by IHC. **(E)** Western blotting results of EPHA2 in EC tissues. G1 I : Grade 1, stage I, endometrioid; G2 II : Grade 2, stage II, endometrioid; G3 III : Grade 3, stage III, endometrioid. **(F)** growth curves after using EPHA2 inhibitor on Ishikawa.

To validate our experimental results from phosphorylation mass spectrometry, we first performed CO-IP experiments to verify the endogenous binding of DUSP1 and EPHA2 in EC. Results as shown in **Figure 7A**, EPHA2 protein could be detected in the IP group incubated with DUSP1 antibody, which proved the protein binding between EPHA2 and DUSP1. Changes of the phosphorylation level of EPHA2 was detected in DUSP1-knockdown and DUSP1 overexpressing EC cells using an antibody that cross recognizes Ser899 phosphorylated EPHA2, while the expression level of total EPHA2 protein was not significantly changed (**Figure 7B, C**).

### Protein expression level of EPHA2 detected by IHC and WB

The protein expression level of EPHA2 was observed by IHC and WB in representative samples. EPHA2 showed homogeneous staining of the cytoplasm in EC samples and the protein expression level of EPHA2 was lower in patients diagnosed with Grade 1 than patients diagnosed with Grade 3 or Grade 2. Representative images are shown in **Figure 7D, E**.

### Effect of EPHA2 inhibitor on endometrial carcinoma

To explore the effect of EPHA2 on endometrial cancer growth, we used the specific EPHA2 inhibitor ALW-II-41-27. The drug concentrations were set at 0 μM, 0.025 μM, 0.05 μM, 0.1 μM, 0.125 μM, 0.25 μM, 0.5 μM, 1 μM, 2 μM, 4 μM, and 8 μM. After incubation for 48 hours, the calculation of cell viability and IC50 values was presented in **Fig 7F**. The IC50 value was 1.258 μM in normal Ishikawa cell line compared to 0.202 μM in Ishikawa with overexpressed EPHA2. These results indicated that overexpression of EPHA2 made EC cell lines more sensitive to ALW-II-41-27. Concentration of 1 μM EPHA2 inhibitor was also used to observe the changes of cell proliferation ability in both untreated and overexpressed EPHA2 Ishikawa. ALW-II-41-27 could significantly inhibit the proliferation of Ishikawa, and the inhibitory effect is more obvious in overexpressed EPHA2 Ishikawa at the same concentration (**Fig 7F**).

## Discussion

The function of DUSP1 has been investigated in many different cancers with inconsistent results, but few studies have touched its function in EC. Our previous study of 113 EC patients showed that DUSP1 low-expression was significantly correlated with advanced stage, higher grade tumor and myometrial invasion 4. In this study, we conducted a deeper investigation on molecular function of DUSP1 and confirmed that DUSP1 could affect tumor apoptosis, growth and invasion in EC using three EC cell lines originated from patients with different tumor stages and pathological grades.

By analyzing the genes highly correlated to DUSP1 from the transcriptome data of EC, this study screened out some transcript factors that may have upstream and downstream regulation relationship with DUSP1, and most of these genes belong to AP-1 or AP-1 expression network. Changes in the expression level of these genes after knockdown and overexpression of DUSP1 suggested that DUSP1 can regulate the expression of AP-1 and its co-expression network related factors. This regulation is achieved by changing the activity of ERK / MAPK pathway through the dephosphorylation of ERK by DUSP1. The analysis of DUSP1 promoter sequence found several AP-1 binding sites. Using AP-1 inhibitor confirmed that AP-1 is the upstream regulator of DUSP1.

The DUSP family consists of 25 genes 22, and DUSP1 is the first discovered member of the DUSP family and can inhibit the biological activities of 3 MAPK pathways through dephosphorylation 23. Activation of MAPK pathways via protein phosphorylation is related to the progression and malignancy of EC 24, and the dephosphorylation on MAPK also plays a key role in inhibiting tumor progression. DUSP1 could target a specific MAPK pathway in different kinds of tumors. In prostate cancer, DUSP1 efficiently dephosphorylated JNK 11; in hepatocellular carcinoma, DUSP1 mainly inhibits the p38/MAPK pathway 12; and in this study, DUSP1 deficiency promoted EC progression via the ERK/MAPK pathway.

The relationship between DUSP1 and AP-1 network was reported in breast cancer 14. Their research was limited to expression correlation and did not investigate any mutual regulation relationship, but did not clarify the relationship between DUSP1 and the AP-1 network.

Our data demonstrate a positive feedback regulatory loop between DUSP1 and AP-1, which plays an important role in inhibiting the growth and invasion of EC. AP-1 transcription factor is assembled from homo- or heterodimers of JUN, FOS, ATF and MAF family proteins to activate or repress its target genes. AP-1 proteins are involved in regulation of a variety of cellular processes, including proliferation, differentiation, apoptosis, and migration. Whether it positively or negatively regulates a specific target gene is dependent upon the composition and abundance of dimers, post-translational regulation, and interaction with other co-regulatory proteins. The effects of AP-1 could be oncogenic or tumor-suppressor depending on the cell type and its differentiation state, genetic background and tumor stage 13.

As we know, various molecules in the cell form a complex system, which contains many negative feedback and positive feedback regulatory systems to maintain the normal function of cells. Generally, negative feedback can correct and weaken the control information, so as to reduce the errors and fluctuations. While positive feedback strengthens the control information and amplifies the control function, which usually exists in the process of cell differentiation and cell cycle transition 25. Our previous studies have showed a higher DUSP1 expression in well differentiated grade 1 tumors, but lower in poorly differentiated grade 3 tumors 4. Thus, we speculated that DUSP1 may control tumor differentiation through DUSP1-ERK/MAPK-AP1 feedback loop. This presumption was supported by the following studies. AP-1 component JUNB was reported to play an important role in regulatory T cell differentiation 26. ZFP36, the co-regulating network member of AP-1 has been reported to be associated with mammary differentiation 27. Activation of MAP kinases and AP-1 were observed during osteoblast differentiation stimulated by corosolic acid 28 and baicalein 29.

However, the specific mechanism of how MAPK regulates all AP-1 network genes is not clear. Previous studies have shown that ERK/MAPK can phosphorylate JUN to reduce its degradation, enhance its stability and increase its transcriptional activity 30. Phosphorylation of other members of the AP-1 complex (e.g., Fra1, Fra2) by MAP Kinase has also been reported. 19-21 Since most members of the co-expression network are transcriptional factors, we speculated that the activated transcription factor members activate other members of the network, resulting in an increase in the expression level of the entire network.

In addition, we also studied the protein modification of DUSP1. While parallel reaction monitoring (PRM) verification based on mass spectrometry is a targeted quantitative technology, it only scans specific target signals and can realize accurate quantification of target proteins and modification sites. Therefore, our research group adopted phosphorylation 4D label-free quantitative proteomics technology based on the new generation ion mobility mass spectrometry (IMMS) to carry out the research. Many new dephosphorylation target proteins of DUSP1 were found. Among them EPHA2 is one of the most important targets, and its interaction with DUSP1 is verified with Co-IP experiment. Changes in the phosphorylation level of EPHA2 were detected in DUSP1-knockdown and DUSP1-overexpressing EC cells.

The implementation of a biological function depends on the cooperation of protein complex members and the synergistic effect of entire biological pathway. DUSP1 targets many unexplored proteins except MAPK pathway, suggesting that the DUSP1 regulatory network might involve other important components, which needs further research. As this regulatory network is associated with EC progression and tumor differentiation, targeting this feedback loop might be an effective anti-cancer strategy to control tumor growth, reverse the low differentiation state or maintain a high differentiation state, and prevent tumor invasion and metastasis.

Previous studies on DUSP1 were limited to its dephosphorylation of MAPK pathway members and its effect on tumor proliferation and progression. This current study not only expanded the understanding of the downstream molecular pathway of DUSP1, but also revealed the upstream regulatory factors of DUSP1. More importantly, this study confirmed that there is a positive feedback regulatory loop between DUSP1 and AP-1 connected by MEK/MAPK pathway. In addition, our pioneering phosphproteomic study in DUSP1-overexpression EC cells demonstrates that DUSP1 could dephosphorylate EPHA2 gene through the ser899 site of EPHA2. This feedback regulatory network has implications in cell differentiation and may provide an insight in developing new therapy strategies.

## Figures and Tables

**Table 1:** Sequences of primers and siRNAs.

Gene	Sequence (5'-3')
Primers	
ATF3-F	ACCGTTAGGATTCAGGCAGC
ATF3-R	TCACTCCACATCCCCTACGA
BHLHE40-F	ACGGAGACCTACCAGGGATG
BHLHE40-R	GGTGCACTTGCTTACCTTGC
DUSP1-F	AGGACAACCACAAGGCAGAC
DUSP1-R	CTCGTCCAGCTTGACTCGAT
EGR1-F	CCTTCAACCCTCAGGCGG
EGR1-R	GAGTGGTTTGGCTGGGGTAA
EPHA2-F	GCAAGGAAGTGGGACCTGATG
EPHA2-R	CTCAGCCTCTCCTCGGTACA
FOS-F	CTTACTACCACTCACCCGCA
FOS-R	AGTGACCGTGGGAATGAAGT
FOSB-F	GAGACTACGACTCCGGCTCC
FOSB-R	TCCTGGCTGGTTGTGATCG
GAPDH-F	GGAGTCCACTGGCGTCTTCA
GAPDH-R	GTCATGAGTCCTTCCACGATA
JUN-F	GAGCTGGAGCGCCTGATAAT
JUN-R	CCCTCCTGCTCATCTGTCAC
JUNB-F	AACAGCCCTTCTACCACGAC
JUNB-R	CAGGCTCGGTTTCAGGAGTT
JUND-F	CCCCCTTCGGTTCTTTCGAC
JUND-R	AAACAGAAAACCGGGCGAAC
MAFF-F	GGACCAGGAGGACGGTCT
MAFF-R	GTGTTCTCGCTCAGCTCTCG
NR4A1-F	CTGGATACACCCGTGACCTC
NR4A1-R	AGGCAGATGTACTTGGCGTT
RGS1-F	ATTGAGTTCTGGCTGGCTTGT
RGS1-R	AGATTCTCGAGTGCGGAAGT
ZFP36-F	AAGGGAGGCAATGAACCCTC
ZFP36-R	AACGGCTTTGGCTACTTGCT
SiRNAs	
DUSP1 Si1-F	GCCAUUGACUUCAUAGACUTT
DUSP1 Si1-R	AGUCUAUGAAGUCAAUGGCTT
DUSP1 Si2-F	GCUUACCUUAUGAGGACUATT
DUSP1 Si2-R	UAGUCCUCAUAAGGUAAGCTT
DUSP1 Si3-F	GCAUCACUGCCUUGAUCAATT
DUSP1 Si3-R	UUGAUCAAGGCAGUGAUGCTT
EPHA2 Si1-F	CCGUCCGUGUCUACUACAATT
EPHA2 Si1-R	UUGUAGUAGACACGGACGGTT
EPHA2 Si2-F	GCGAGUGUGAGGAAGGCUUTT
EPHA2 Si2-R	AAGCCUUCCUCACACUCGCTT
EPHA2 Si3-F	CCUGGCCAACAUGAACUAUTT
EPHA2 Si3-R	AUAGUUCAUGUUGGCCAGGTT

**Table 2:** Co-expression analysis of DUSP1 in EC.

Correlated Gene	Cytoband	Spearman's Correlation	p-Value	q-Value
ZFP36	19q13.2	0.847	5.24E-^50^	1.04E-^45^
FOS	14q24.3	0.846	1.03E-^49^	1.04E-^45^
FOSB	19q13.32	0.778	3.26E-^37^	2.18E-^33^
EGR1	5q31.2	0.757	3.52E-^34^	1.77E-^30^
ATF3	1q32.3	0.754	8.97E-^34^	3.60E-^30^
NR4A1	12q13.13	0.679	3.10E-^25^	1.04E-^21^
JUNB	19p13.13	0.659	2.21E-^23^	6.34E-^20^
CSRNP1	3p22.2	0.606	4.11E-^19^	1.03E-^15^
RGS1	1q31.2	0.604	5.50E-^19^	1.23E-^15^
JUN	1p32.1	0.591	4.92E-^18^	9.88E-^15^
TCIM	8p11.21	0.589	6.95E-^18^	1.27E-^14^
BTG2	1q32.1	0.584	1.49E-^17^	2.49E-^14^
CCN1	1p22.3	0.582	2.06E-^17^	3.19E-^14^
KLF6	10p15.2	0.578	3.40E-^17^	4.88E-^14^
SOCS3	17q25.3	0.575	5.85E-^17^	7.83E-^14^
IL6	7p15.3	0.573	8.25E-^17^	1.04E-^13^
NR4A3	9q31.1	0.568	1.69E-^16^	2.00E-^13^
MAFF	22q13.1	0.566	2.10E-^16^	2.34E-^13^
RHOB	2p24.1	0.558	7.55E-^16^	7.98E-^13^
GPR183	13q32.3	0.556	9.70E-^16^	9.28E-^13^
BHLHE40	3p26.1	0.556	9.71E-^16^	9.28E-^13^
DUSP5	10q25.2	0.554	1.21E-^15^	1.11E-^12^
PPP1R15A	19q13.33	0.535	1.66E-^14^	1.45E-^11^
GEM	8q22.1	0.522	9.57E-^14^	8.01E-^11^
SIK1	21q22.3	0.52	1.13E-^13^	9.05E-^11^
EGR3	8p21.3	0.505	7.48E-^13^	5.78E-^10^
